# A case of extraperitoneal stoma-associated internal hernia after abdominoperineal resection

**DOI:** 10.1186/1477-7819-12-141

**Published:** 2014-05-06

**Authors:** Yuichiro Yokoyama, Kazushige Kawai, Shinsuke Kazama, Satomi Yoneyama, Junichiro Tanaka, Toshiaki Tanaka, Tomomichi Kiyomatsu, Hiroaki Nozawa, Takamitsu Kanazawa, Hironori Yamaguchi, Soichiro Ishihara, Eiji Sunami, Joji Kitayama, Toshiaki Watanabe

**Affiliations:** 1 Division of Surgical Oncology, Department of Surgery, Faculty of Medicine, University of Tokyo, 7-3-1 Hongo, Bunkyo-ku, Tokyo 113-8655, Japan

**Keywords:** Abdominoperineal resection, Extraperitoneal stoma, Internal hernia, Rectum

## Abstract

Published reports concerning internal hernias after extraperitoneal stoma construction are scarce. In our present report, we describe the case of a 56-year-old man who was referred to our hospital for the treatment of rectal cancer. He underwent abdominoperineal resection of the rectum with sigmoidostomy using an extraperitoneal route. On the ninth postoperative day, the patient experienced sudden and intense abdominal pain and was diagnosed with strangulation of the small intestine due to a stoma-associated internal hernia. Therefore, an emergency laparotomy was performed. The surgical findings showed that the small intestine protruded through the space between the sigmoid colon loop and the abdominal wall in a cranial-to-caudal direction. The strangulated portion of the small intestine was recovered, and the orifice of herniation was closed. No recurrence of internal herniation was observed during the follow-up period.

## Background

Abdominoperineal resection (APR) was first described in 1908 by Miles [1]. Although sigmoidostomy construction through the abdominal route was the original method described by Miles, many cases of stoma-associated bowel obstruction complications, such as parastomal hernia and internal hernia, have been reported since then [2-4]. In 1958, 50 years after the Miles’s original publication describing APR, Goligher reported the use of an extraperitoneal stoma, a novel technique of stoma construction to avoid bowel obstruction complications [5]. Indeed, after Goligher’s report, the induction of the extraperitoneal stoma markedly reduced the incidence of stoma-associated bowel obstruction complications. We found only one other previous report of extraperitoneal stoma–associated internal hernia in the literature [6]. However, although it is quite rare, internal hernia after stoma construction via an extraperitoneal route remains a possible complication. Thus, in our present report, we describe the case of a patient in whom a portion of the small intestine protruded through the narrow gap between the sigmoid colon and the abdominal wall, with subsequent strangulation after extraperitoneal sigmoidostomy during APR.

## Case presentation

**Figure 1:**
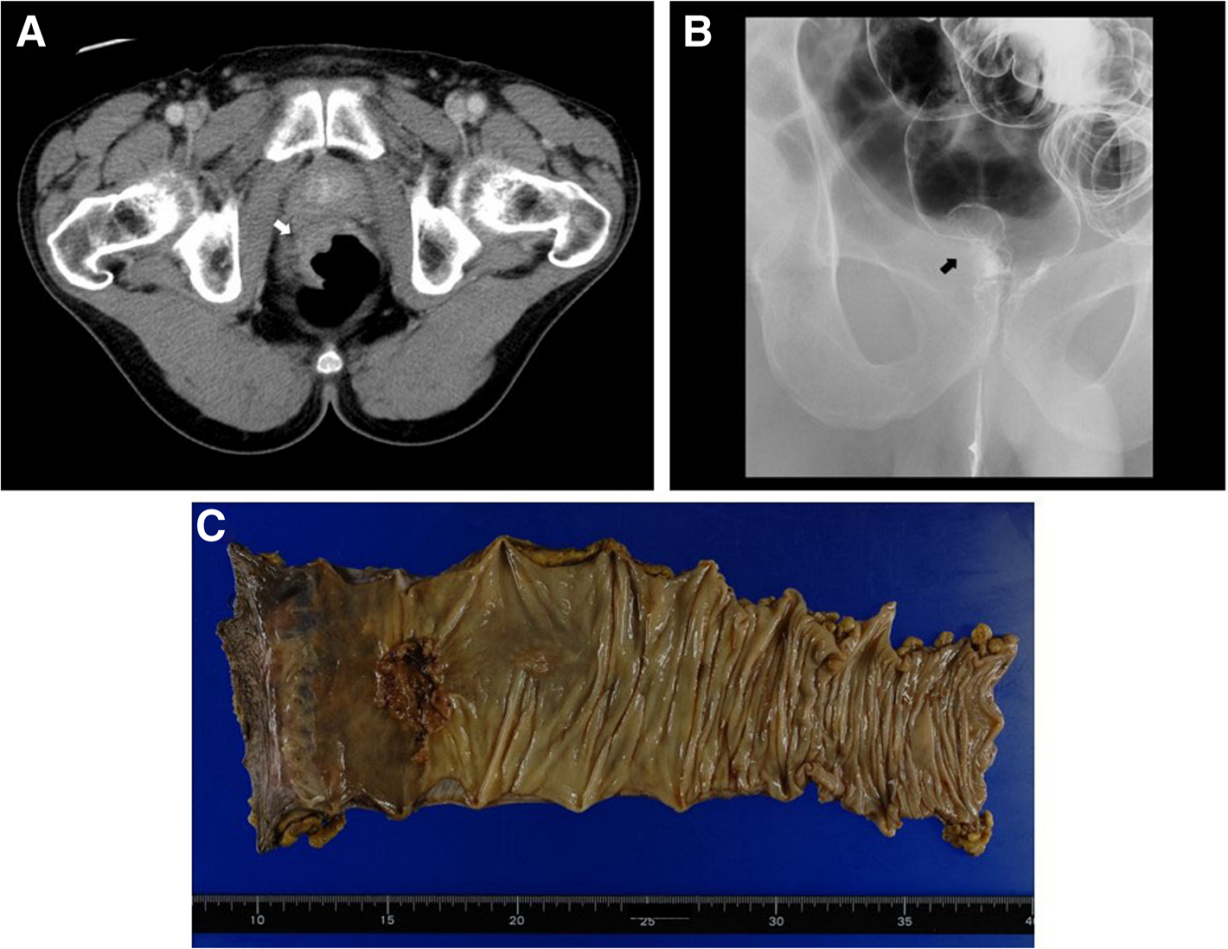
**Preoperative computed tomography, barium enema and gross appearance of the resected tumor specimen.** Preoperative computed tomography scan **(A)** and barium enema **(B)** showing the presence of rectal cancer at the anterior wall of the lower rectum, close to the anal canal. **(C)** The resected cancer specimen had a diameter of 35 mm.

A 56-year-old man who had a positive result on a fecal occult blood test was referred to our hospital. Advanced rectal cancer 2 cm from the anal verge was detected by colonoscopy and barium enema (Figures 1A and 1B). Lymph node metastases on the right and left sides of the internal iliac artery and lung metastases on both sides were suspected upon inspection of computed tomography (CT) and [^18^ F]-fluorodeoxyglucose positron emission tomography scans. The patient underwent APR of the rectum with lateral lymph node dissection on both sides (Figure 1C). While performing lateral lymph node dissection, we partially resected the retroperitoneum on the pelvic wall. After sigmoidostomy through an extraperitoneal route, we performed closure of the retroperitoneum with knot sutures.

**Figure 2:**
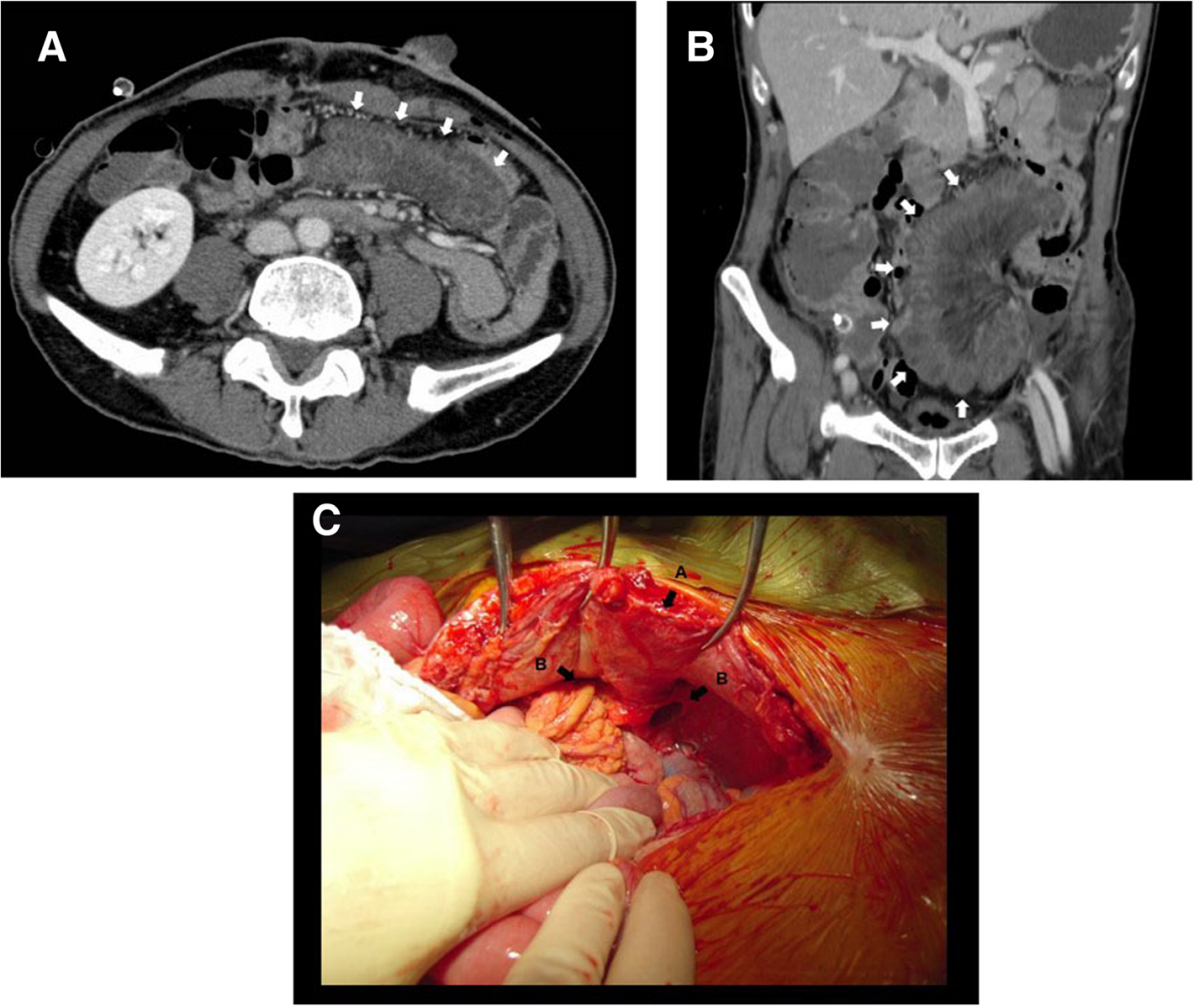
**Emergency computed tomography and laparotomy findings after reduction of the herniated intestine. (A)** and **(B)** After the onset of acute abdominal pain, the emergency computed tomography scan shows a loop of small intestine obstructed by the abdominal wall and sigmoid colon that resulted in sigmoidostomy (white arrows). The wall of the obstructed intestine is edematous and less enhanced. **(C)** The left side of the photograph is indicative of the cranial direction, and the right side shows the caudal direction. Sigmoidostomy is indicated by the black arrow marked A, and the route of herniation through the space lateral to the sigmoid colon is indicated by the black arrows marked B.

Although the patient’s postoperative course was uneventful, he experienced a sudden onset of intense abdominal pain on the ninth postoperative day. Emergency CT showed the loop of a poorly enhanced edematous small intestine that was strangulated by the sigmoid colon and abdominal wall (Figures 2A and 2B). Immediately after we made the diagnosis of strangulation of the small intestine caused by the stoma-associated internal hernia, an emergency laparotomy was performed. The surgical findings showed that the sutured mesocolon and lateral peritoneum were separated and that a segment of the small intestine from the terminal ileum with a length of 110 to 180 cm passed through the space between the sigmoid colon loop and the abdominal wall in a cranial-to-caudal direction (Figure 2C). Although the segment of protruded small intestine showed moderate ischemic change, the strangulated segment was reduced and its color quickly normalized. Both the cranial and caudal sides of the hernia orifice were closed by suture to prevent recurrent herniation. The patient was discharged on the 28th day after the second operation without any further complications. No recurrence of herniation was observed until the patient died 21 months after surgery owing to recurrent cancer and progression.

## Discussion

Although intestinal obstruction has previously been reported to occur in 3.5% to 7.2% of cases after colostomy [2-4], the majority of them were caused by postoperative adhesions [2]. The proportion of internal hernia associated with extraperitoneal stoma in the postoperative ileus is quite small, with only one precedent report in the literature [6]. Although it is unlikely, this complication is still possible, as illustrated in our present report. Although closure of the lateral space by suture is helpful to avoid this complication, complete closure can be difficult occasionally.

**Figure 3:**
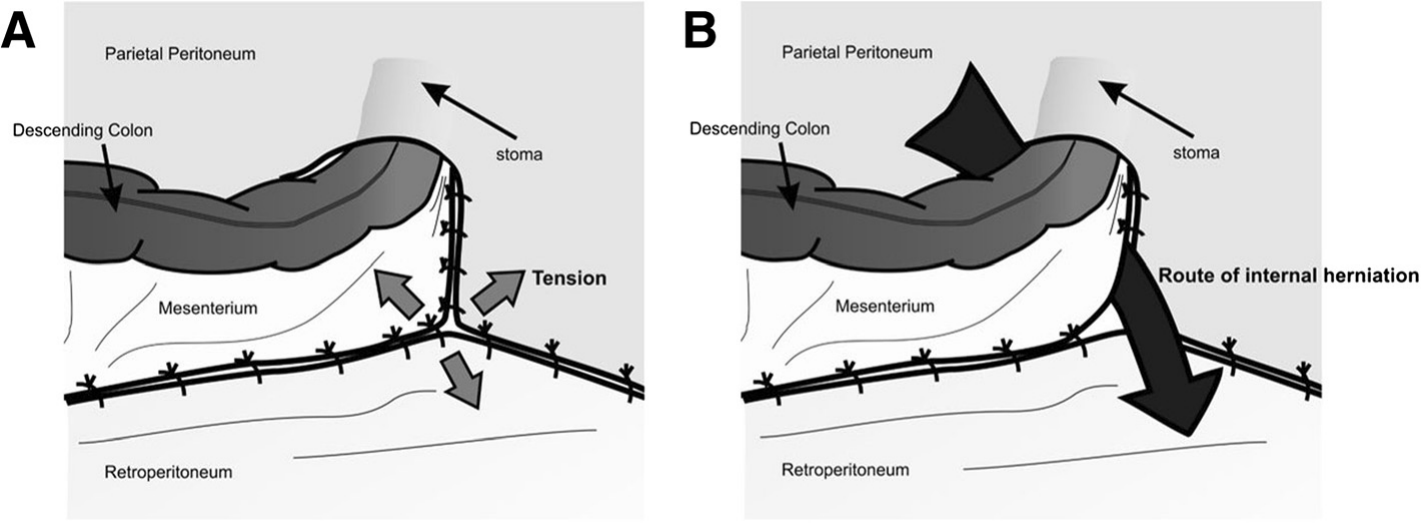
**Schemes illustrating the internal hernia. (A)** This illustration shows reperitonealization after abdominoperineal resection of the rectum by sigmoidostomy through an extraperitoneal route. **(B)** This drawing shows the route of internal herniation.

In the present case, because metastasis to the lymph nodes surrounding the internal iliac artery was suspected, we performed lateral lymph node dissection, which shortened the pelvic wall peritoneum. Although we managed to close the retroperitoneum, the closure was under strong tension, and the closure was disrupted at the time of the second surgery (Figures 3A and 3B). In patients in whom the closure of the peritoneal defect is performed under tension, as in our patient, the closed caudal herniation orifice may become disrupted postoperatively. Therefore, it is important to completely close the cranial hernia orifice by suturing the lateral peritoneum edge to the descending and sigmoid colon to avoid an internal hernia. Indeed, we closed the cranial orifice as well as the caudal orifice during the second surgery, and no recurrence of internal herniation was observed during the follow-up period.

The development of laparoscopic surgery in recent years has contributed to the difficulty in closing the space lateral to the sigmoid colon. Laparoscopic colectomy, including APR, has recently become very popular, and sigmoidostomy is constructed through an intraperitoneal route in many institutions. Although the laparoscopic extraperitoneal colostomy technique has been developed to avoid parastomal herniation [7-9], it requires additional operating time. Moreover, if the colostomy is constructed through an extraperitoneal route, the laparoscopic closing of the lateral space of the colostomy by suture is very difficult. Additionally, postoperative adhesion observed with laparoscopic surgery is decreased with the use of this technique, which may lead to an increase in the occurrence of postoperative internal hernia [10,11]. Therefore, along with the popularity of laparoscopic APR, the incidence of stoma-associated internal hernia is expected to increase.

## Conclusion

Internal hernia associated with extraperitoneal stoma is a rare but possible complication that should be considered when treating a patient with ileus caused by small-intestine obstruction after APR.

## Consent

Written informed consent was obtained from the patient for publication of this case report and any accompanying images. A copy of the written consent is available for review by the Editor-in-Chief of this journal.

## Abbreviations

APR: Abdominoperineal resection; CT: Computed tomography.

## Competing interests

The authors declare that they have no competing interests.

## Authors’ contributions

YY conceived of the study, collected data and drafted the manuscript. KK conceived of the study and corrected and revised the manuscript. SK, SY, JT, TT, TK, HN, TK and HY helped in drafting the manuscript. SI, ES, JK and TW corrected and revised the manuscript. All authors read and approved the final manuscript.
